# Enabling structural resilience of street-involved children and youth in Kenya: reintegration outcomes and the Flourishing Community model

**DOI:** 10.3389/fpsyg.2023.1175593

**Published:** 2023-08-23

**Authors:** Michael L. Goodman, Sarah E. Seidel, Andrew Springer, Aleisha Elliott, Christine Markham, Hani Serag, Philip Keiser, Ben Raimer, Lauren Raimer-Goodman, Christine Gatwiri, Kelvin Munene, Stanley Gitari

**Affiliations:** ^1^Department of Internal Medicine, The University of Texas Medical Branch, Galveston, TX, United States; ^2^Sodzo International, Houston, TX, United States; ^3^The University of Texas Health Science Center at Houston, Houston, TX, United States; ^4^Texas AHEC East, The University of Texas Medical Branch, Galveston, TX, United States; ^5^School of Public and Population Health, The University of Texas Medical Branch, Galveston, TX, United States; ^6^Office of the President, The University of Texas Medical Branch, Galveston, TX, United States; ^7^Community-based Clinics, The University of Texas Medical Branch, Galveston, TX, United States; ^8^Sodzo Kenya, Maua, Kenya

**Keywords:** street-involved children and youth, resilience, socio-ecological frameworks, reintegration, Kenya

## Abstract

**Introduction:**

Millions of children and youth live on city streets across the globe, vulnerable to substance use, abuse, material and structural neglect. Structural resilience, the re-establishment of access to structural goods within a society such as housing, education, and healthcare following some interruption, provides an orientation for research and interventional efforts with street-involved children and youth (SICY). Further, a structural resilience framework supports organizing interactions between levels and sectors of a socio-ecology.

**Methods:**

Following the expressed interests of Kenyan SICY, and consistent with emerging policy interests at national and global levels, we assess reintegration trajectories of Kenyan SICY (*n* = 227) participating in a new program intervention and model. The intervention combines two coordinated, parallel programs – one focused on the rescue, rehabilitation, reintegration and resocialization of SICY, and the other focused on empowering families and communities to provide better care for children and youth who are reintegrating from life on the streets to the broader community. Data were collected and analyzed from multiple stages across SICY involvement with the intervention.

**Results:**

We found 79% of SICY participants reintegrated with the broader community, and 50% reintegrated with families of origin and returned to school. Twenty-five percent of participants reintegrated to a boarding school, polytechnical school, or began a business. Probability of reintegrating successfully was significantly improved among participants whose families participated in the family- and community-oriented program, who were younger, with less street-exposure, expressed more personal interests, and desired to reintegrate with family.

**Discussion:**

To our knowledge, these are the first quantitative data published of successful reintegration of SICY to the broader, non-institutionalized community in any low- or middle-income country. Future research should (1) identify factors across socio-ecological levels and sectors contributing to health and developmental outcomes of reintegrated children and youth, (2) mechanisms to support SICY for whom the interventional strategy did not work, (3) methods to prevent street-migration by children and youth, and (4) system development to coordinate follow-up and relevant investment by institutions, organizations and community leaders to continue reintegration work.

## 1. Background

### 1.1. Overview of challenges facing street-involved children and youth

The United Nations Children’s Fund has previously published estimates that tens of millions of children live on city streets globally, separated from adult caregivers (UNICEF, 2005). A notoriously difficult population to enumerate, street-involved children and youth (SICY) are vulnerable to multiple forms of abuse on the streets, human trafficking, substance misuse, and failure to thrive (Mathur et al., 2009; Nada and El Daw, 2010; Malindi and MacHenjedze, 2012; Pullum et al., 2012; Embleton et al., 2013; Aransiola and Zarowsky, 2014).

Published estimates of the number of Kenyan SICY range from 46,639 to 300,000 (Sorber et al., 2014; Street Families Rehabilitation Trust Fund, 2018). The study producing the lower estimate, released by the Kenyan Ministry of Labour and Social Protection in 2018, faced multiple limitations undermining its reliability – such as distrust between enumerators and children, budget over-runs due to the need for increased security, language barriers between interviewers and children, and criminal cartels who interfered with the underfunded study (Street Families Rehabilitation Trust Fund, 2018). The higher estimate is regularly cited as authoritative in peer-reviewed literature; however, it was released as a report from a news agency in 2007, citing the estimates of experts without peer-reviewed analysis or methodological transparency (IRIN, 2007).

### 1.2. The Socio-ecological context of street-involved children and youth in sub-Saharan Africa

**FIGURE 1:**
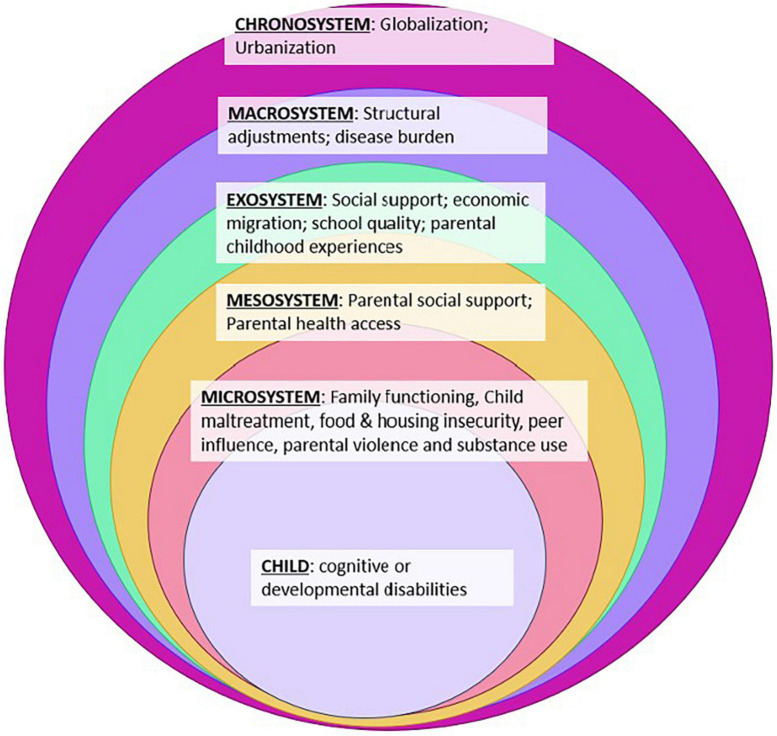
Non-exhaustive socio-ecological factors influencing street-migration of children.

The phenomenon of children living on the street is a multi-level problem, understood best through Bronfenbrenner’s socio-ecological model. Figure 1 displays a non-exhaustive compilation of known factors associated with street-migration among children. Across time (chronosystem), industrialization and dominant global economic development models contribute to urbanization (Patil, 2014). According to United Nations data, the percentage of population living in urban areas in Eastern Africa increased fourfold between 1950 and 2020 – equal to the growth in Kenya (United Nations, Department of Economic and Social Affairs, Population Division [UNDESAPD], 2018). Across sub-Saharan Africa, including in Kenya, urbanization followed railways placed by colonizing European country governments – a trajectory that continued after railways became less utilized (Jedwab and Moradi, 2016; Jedwab et al., 2017a). At the beginning of the 20th century, sub-Saharan Africa contained only about 50 cities with 10,000 or more inhabitants. By 2010, the number of cities with at least 10,000 inhabitants grew to almost 3,000 (Jedwab et al., 2017b).

The emergence of cities, new economic opportunities and political economies influence macro-, exo-, meso-, and micro-systems impacting risk of street migration among children. One example of the impact urbanization and new migration routes have on communities and families is the early geographic spread of HIV – which followed economic routes created by colonizing forces (Faria et al., 2014). While it is difficult to trace the numerical rise of SICY across colonizer-induced dynamics, urbanization is a necessary element for children to sleep on city streets. Further exacerbating disruptive inequities due to forced participation in the globalized economy, the structural adjustment programs inspired by the Washington Consensus and championed by the great powers in the late 1980s exacerbated the relative standing of rural communities, women and children with respect to health outcomes, income, education, and social cohesion (Ahmed and Lipton, 1997; Craig and Porter, 2005; Thomson et al., 2017; Forster et al., 2020).

### 1.3. Policy environment related to interventions with street-involved children and youth

There is a dire need for evidence-informed programmatic and policy interventions to support the wellbeing and self-determination of SICY. In 2010, the United Nations General Assembly adopted a resolution calling for member states to shift from placement of children in long-term care facilities toward reintegration of separated children (United Nations General Assembly [UNGA], 2010). In 2012, the United Nations High Commissioner for Human Rights published a report on “the protection and promotion of the rights of children working and/or living on the street.” The report recognized governments as primary duty-bearers to meet the obligation of having the rights of children respected and fulfilled. The UN High Commission report required States to present legislation to mandate municipal policies and resource-coordinated interventions for SICY (Office of the High Commissioner for Human Rights [OHCHR], 2012). The United Nations Convention on the Rights of the Child issued a general comment in 2017 aiming to provide comprehensive, authoritative guidance to States toward a holistic, rights-based approach to prevent street-migration of children and ensure a continuum of care for SICY to develop their fullest potential (UN Committee on the Rights of the Child, 2017).

Kenya is one of many low- and middle-income countries working to address the social and health challenges confronting SICY. In 2022, the national government of Kenya released its National Care Reform Strategy for Children endorsing reforms to (1) prevent family separation and promote family strengthening, (2) support alternative care and transition away from institutional care, and (3) trace, reintegrate and transition to family- and community-based care (Kenya Ministry of Labour and Social Protection, 2022). Kenya’s national plan for care reform includes efforts to defund institutionalization of children, a plan that is supported by good evidence (e.g., Lionetti et al., 2015; Lyneham and Facchini, 2019). However, due to the paucity of evidence, neither the UN resolution nor the Kenya Care Reform strategy provides evidence to support the perspective that SICY can be successfully reintegrated with families of origin or foster families. A 2016 global review of literature presenting interventional effectiveness of programs designed to reintegrate SICY found no studies measuring inclusion and reintegration of SICY anywhere in the world, and no studies exploring interventional outcomes of programs in low- or middle-income countries (Coren et al., 2016). Evidence-informed strategies are required to ensure that children living on the streets are not simply placed back in abusive or unstable families they fled when they initially migrated to the streets.

### 1.4. Study interventional context

**FIGURE 2:**
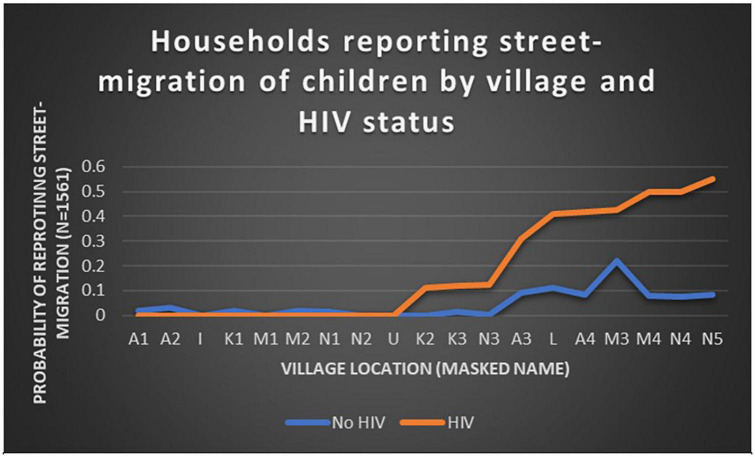
Variation in street-migration by village and parental HIV status within three Kenyan sub-counties. Data from Goodman et al. (2017).

A team of public health researchers, community leaders and social workers, our own work with SICY began in 2012 in response to local concerns about the growing number of children who were migrating to the streets of Meru County, Kenya (Seidel et al., 2017, 2018). Figure 2 illustrates the heterogeneity of households reporting street-migration of children between communities within three sub-counties in Meru County, stratified by HIV-status. Statistically significant variation in probability of reporting a child lives on the street differs by HIV-status of adults in the household and village location (Goodman et al., 2016). Mixed methods research reveals multiple community- and family-level predictors of street-migration – including maternal childhood adversities, maternal years of schooling, family cohesion, financial resources, parental mental health, substance use, and maternal social support (Goodman et al., 2017). Learning disabilities appear at higher rates among street-involved children than within the broader population, indicating child-level differences that may contribute to street-migration risk (Ward and Seager, 2010).

#### 1.4.1. Risk of stigma from non-contextualized interventions

Despite the complex socioecology of street-migration among children, many SICY experience high rates of stigma related to life on the streets (Rivenbark et al., 2018; Gayapersad et al., 2020). Often interventions to assist vulnerable children and promote child resilience are not adequately informed by a socio-ecological model and may risk reinforcing an individualistic view of children’s resilience capacities (van Breda and Theron, 2018). An individualistic focus is particularly unhelpful, and blames the victims who are children facing enormous socio-ecological challenges. Other interventions with stigmatized populations report that an individualistic-focus places the blame and responsibility for one’s stigmatized condition on individuals and thereby reinforces the stigmatized label (Täuber et al., 2018). Therefore, we sought to develop a socioecological model to support the reintegration of SICY, pursuant to their expressed desires and consistent with current Kenyan policy.

### 1.5. Study interventional design

Interventions must operate within their available scope of influence; within the communities of Meru County, Kenya, our intervention aims to address modifiable constructs and factors at two levels of the ecological model: (1) the children identified as living on the street and (2) the families of these children (Goodman et al., 2020). Through iterative action-reflection cycles, we developed a “4R + P” model to support children living on streets of three sub-counties in Meru County, Kenya – Rescue, Rehabilitation, Reintegration, Resocialization, and Prevention (Table 1). Program goals are to: (1) help interested SICY find their way off city streets (Rescue); (2)help rehabilitate SICY by providing a nurturing environment to detoxify from substance use, anti-social street behaviors, and reconnect with family, community mentors, and schools (Rehabilitation); (3) connect former SICY with their families of origin whenever possible and support these families to provide continuous on-going nurturing (Reintegration); (4) help children and youth form new identities that support their future roles as community members and leaders through caring for crops, animals, gaining marketable skills and re-initiating school attendance (Resocialization); and (5) strengthen families and communities to prevent the migration of children to the street and ensure children have secure and nurturing environments within which to grow and develop (Prevention).

**TABLE 1:** 4R + P model for working with street-involved children and youth.

Rescue	Rehabilitation	Reintegration	Resocialization	Prevention
Build rapport with children living on the streets	Provide temporary secure shelter, stimulation and support for child	Transfer child and care to identified family member when possible	Utilize practical skills of self-care, care for animals and crops	Increase social, economic, health, and educational resources at village-level
Identify children interested in leaving life on the streets	Cease use of substances, and learn coping and prosocial skills through group and individual counseling	Transfer child to boarding school or polytechnic school if necessary	Demonstrate school readiness and attend nearby school	Cultivate nurturing “flourishing” communities to support member families and children
Work with District Children’s Office to secure approval to assume care for child	Form new role/social identity as “child of promise”	Provide on-going follow-up to identify and rectify challenges	Re-develop connections with family members when possible	

#### 1.5.1. Introducing the Watoto wa Ahadi Rescue Center

Our strategy to support the rescue, rehabilitation and resocialization of SICY is called the “Watoto wa Ahadi Rescue Center” or “Children of Promise Rescue Center.” The program has gained the shortened name “ARC.” The ARC is based on 79-acres owned by the Methodist Church of Kenya and has been developed to include housing for 50 children at one time, staff dormitories, kitchen and dining hall, remedial school building, community meeting place, farm animals and crops – including a kitchen garden. Recruitment to the ARC initially began with the expectation that SICY would spend up to 2 years in the center, in part to provide the program time to develop the community-based program (KPJ, more below) within local area communities and SICY’s communities of origin, and in part because there were no data to guide the intervention planning process. It became clear that some children and youth were ready to reintegrate sooner, and provisional support for reintegrating children and youth was thus developed through the community-based element described below. These shifts also coincided with early COVID-19 policy in Kenya that precluded gatherings of more than 10 individuals, thus forcing the program to reintegrate youth more rapidly than before. Currently, some youth are directly reintegrated (spending little to no time at the ARC) if program social workers assess them to be less integrated with street-life and their families to have some capacity to care for them.

#### 1.5.2. Introducing the Flourishing Community model and the KPJ program

Our strategy to reintegrate former SICY, and prevent their migration to the street, requires community engagement. Since 2017, we have been iteratively designing and testing features of the model we now call “Flourishing Communities” (Goodman et al., 2022a). Beginning with the home village of a child identified as living on the street, this program has grown to over 39 villages with over 10,000 weekly participating families (as of December 2022). While the overall model, intended to be generalizable beyond its specific context, is called Flourishing Communities, the program that continues to give rise and clarity to the Flourishing Community model is called “Kuja Pamoja kwa Jamii” (*KPJ*; Swahili for “Come Together for the place where we belong”). An adaptation and expansion of group-based microlending and communal governance approaches, more has been published on the KPJ design, organization and practices (Goodman et al., 2021a) and proposed psychosocial mechanisms (Goodman et al., 2022a).

##### 1.5.2.1. Distinctions and similarities between flourishing and resilience

**FIGURE 3:**
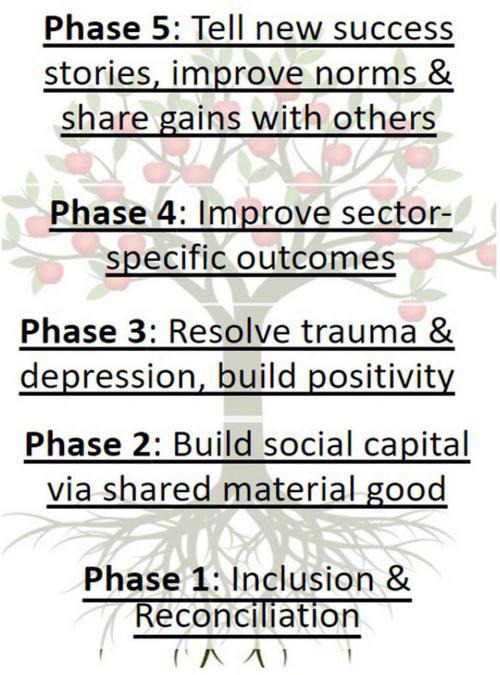
Flourishing Community model.

The terminology “flourishing” was selected deliberately to underscore that while the communities that participate in the program face various challenges from multiple sources, levels, and histories, it is their potential and opportunities for growth that define the program staff’s relationship with them (Goodman et al., 2022a). While resilience is a process in response to adversity, flourishing is a process in response to opportunity. As communities experience both adversity and opportunity, resilient and flourishing processes overlap in lived experience as well as theoretical underpinnings. Yet, the ways in which outsiders frame and engage with communities influences communal self-perceptions and should be carefully considered (Muhammad et al., 2015). We anticipate that self-understandings that prioritize opportunities rather than adversities are more likely to support empowerment and respect human dignity. Definitions of resilience have included both an orientation toward adversity and new found opportunities, but we believe calling the latter definition “flourishing” clarifies this tension and places this work within other broad literature on psychosocial and economic dynamics of human flourishing (Shaw, 2012; VanderWeele, 2017). The Flourishing Community model is represented to program participants through use of the tree (Figure 3), and seeks to enhance structural resilience.

##### 1.5.2.2. Structural resilience

Resilience has been defined many different ways – ranging from intra-individual traits, states, or processes to adaptability of communities or organizations (Pooley and Cohen, 2010). This study approaches resilience as a structural process within a socio-ecology; there are certain socio-ecological positions that must be addressed and resolved before questions of individual-level resilience are posed ethically. At the center of this study is an investigation of “structural resilience” – what enables the possibility of changed structural relations for children and youth living in city streets of Meru County, Kenya. The construct of structural resilience describes the interacting and mutually supporting legal, economic, social, and political structures within a society that ensure equitable access to quality housing, education, and healthcare to promote people’s individual and collected self-determination (adapted from Panter-Brick, 2014). Structural resilience has appeared in scholarly literature occasionally over the past decade, but remains conceptually and operationally under-utilized (Southwick et al., 2014; Manjula and Srivastava, 2022). Within the policy shift toward reintegrating SICY, and children in institutional settings, the concept of structural resilience provides a framework to consider the socio-ecological factors that contribute to sustained reintegration and resocialization of SICY and primary prevention of their street-migration.

#### 1.5.3. The 5-phase scaffolding approach to Flourishing Community

To support the structural resilience of SICY, we developed a two-pronged approach within the overall umbrella we call the “Flourishing Community” model. Programmatically, one prong focuses directly with children who are living in street contexts (the ARC program), and the other prong focuses on families and communities from which these children migrate (the KPJ program).

As displayed in Figure 3, the Flourishing Community model begins with inclusion and reconciliation (Phase 1). Inclusion may refer to inclusion of former SICY, their families, others who do not experience inclusion or require reconciliation with other program members. As roots draw resources into the tree, included and reconciled members of Flourishing Communities bring assets to the community and permit connection. The next phase (Phase 2) involves the establishment of lending groups who convene weekly to exchange $0.20–$0.50 with other members, and thereby generate social capital – expectations of reciprocity, trust, and shared capacity to improve their lives together (c.f. Goodman et al., 2021a,b, 2022b,c). As the trunk supports the structure of the tree, increased economic and social resources support the growth and structure of Flourishing Communities.

The social capital accrued through weekly microfinance participation enables members to address sources of on-going trauma, reduce depression, and build psychological assets like meaning in life, spirituality, curiosity, compassion, and self-compassion (Phase 3) (cf. Goodman et al., 2021b). To support this psychological development, we have created and are testing a novel positive psychology-based curriculum (“Pathways to Flourishing”), integrating insights from interpersonal theories of depression, psychological flexibility, and positive psychology (Kashdan and Rottenberg, 2010; Fredrickson, 2013; Hames et al., 2013; Seligman, 2018). As the xylem in trees carry water and dissolved minerals up from the roots of a tree to the leaves and fruit, positive psychological resources permit individuals and communities to “broaden and build” engagements and cultivate new opportunities and resources (Fredrickson, 2013).

Enhanced economic, social, and psychological resources permit communities to advocate for, and collaborate on, community resources and development across sectors and domains (Phase 4). In practice, this takes the appearance of advocating for and securing new water wells, school buildings, housing support, farming skills, HIV testing, peer support to reduce intimate partner violence, and other areas. The model presents an opportunity to consider how the Sustainable Development Goals may be integrated at the community level (cf. Stafford-Smith et al., 2017). As branches lead from the trunk of a tree in different directions and produce leaves to metabolize energy from the sun and flowers to recruit bees and promote pollination, organized, organic and empowered growth within Flourishing Communities can lead to improvements across multiple domains and support liaising with external resources.

Sustainable community growth and development leaves lasting benefits to future generations and inspires further community-led change within one’s own and in other communities (Phase 5). As fruit indicates the growth of healthy trees and carries seeds to develop other trees and their own fruit, the results of Flourishing Communities improvements across sectors and domains will result in lasting benefits and will inspire other communities. For more extensive discussion of the social psychology and facets of community development of the Flourishing Community model, (please see Goodman et al., 2022a).

Examples of measures reflecting the phases of the Flourishing Community model are presented in Table 2. These data are from on-going program evaluation to understand and inform processes by which the KPJ intervention may impact participants. While we have not yet assessed the interventional model through a randomized control trial, longitudinal data demonstrate evidence of effectiveness for the Flourishing Community model. Global sense of belonging, household monthly income, collective efficacy, compassion, depression, spirituality, HIV-related stigma and harsh child punishment all improve from the baseline (T1) occurring 1-year prior to the follow-up (T2) among active participants. The KPJ program has sufficient enrollment to permit multiple concurrent studies and exploration of measures (see Table 2 data).

**TABLE 2:** Measures reflecting Flourishing Community model, baseline (T1) vs. follow-up (T2) among KPJ participants, Meru County, Kenya.

		T1		T2		
	***N***	**Mean**	**SD**	**Mean**	**SD**	***p*-Value**
Global sense of belonging^t^	60	59.6	−9.1	63.2	−7.9	<0.01
HIV-related stigma*	88	15.4	−5	12.7	−5	<0.001
Monthly household income (USD)	229	29	−30.4	36	−42.2	<0.01
Collective efficacy*	184	5.8	−0.8	6	−0.7	<0.001
Compassion	133	23.4	−2.1	23.9	−1.7	<0.05
Depression	223	0.46	−0.47	0.34	−0.34	<0.001
Spirituality*	118	6.4	−0.68	6.6	−0.63	0.01
Harsh child punishment, past month	229	28.30%	−0.5	19.60%	−0.4	<0.01

Participants (T1) who reported highest values of compassion, spirituality, or collective efficacy and lowest level of HIV-related stigma were removed from the bivariate analysis shown here. Variables are ordered to depict the logic model of the 5-phases of Flourishing Community scaffolding: (1) build belonging/inclusion – here, global sense of belonging, (2) build economic and social resources simultaneously – here, income and collective efficacy, (3) improve mental health and psychological – here, compassion, depression, and spirituality, (4) improve sector specific outcomes – here, parenting; and (5) normative and enduring improvement (not shown). Data from multiple studies currently under peer-review. Asterisk indicates variables where highest or lowest T1 values were removed from analysis to deal with ceiling or floor effects.

^t^Global sense of belonging data come from an adaptation of program to families with HIV, thus comprising a smaller subset of participants.

### 1.6. The current study

This study analyzes exit data from the ARC program to inform probability of “successful” reintegration of former SICY. Outcomes were created following the 4R approach – sustainably rescued and rehabilitated from living on the streets (i.e., the child did not abandon the program early or return to the streets after leaving the program), resocialized (i.e., demonstrates ability to be sustained in an academic- or work-oriented program), and reintegrated (i.e., engaged in suitable activities to promote the child or youth’s continued growth and development in an extended fashion).

#### 1.6.1. Study data context

The ARC program initiated programmatic intervention in April 2016, and its model has evolved since. An open question programmatically, and relevant to the policy enthusiasm for closing long-term charitable child institutions, is how long a child or youth should remain in a transition or rehabilitation facility. The program initially began with a 2-year time horizon for a cohort of children, owing in part to strategic development of the KPJ program envisioned during the second year. Experience operating the program clarified that each case is different, with some children and youth able to return to their home environments much sooner than other children and youth. Alternatively, some SICY are able to be reintegrated directly while spending no time, or only a few days, at the ARC program. The degree to which time duration spent at a transitional facility predicts SICY’s reintegration with families of origin, school attendance, or recidivism to street life has not to our knowledge been reported.

The duration of time a child or youth spends on the streets influences the degree to which that person is socialized into the norms, attitudes and behaviors of street-life. For example, children who spend more time sleeping on the streets are at greater risk of substance use (Goodman et al., 2023). Overall, global prevalence estimates indicate 60% of SICY utilize some form of substance while on the street (Embleton et al., 2013). The degree to which duration on the streets or substance use patterns on the street impact reintegration prospects within sub-Saharan Africa has not been previously reported.

Children and youth face enormous adversity on city streets – encountering economic, emotional, physical and sexual abuse in addition to the social and material deprivations (Mathur et al., 2009). A previous study from Burundi found the number of traumatic life events and violent experiences during the previous 3-months predicted the number of classes attended by SICY at an institutional care facility (Crombach et al., 2014). The degree to which abuse experienced on the streets, which often compounds maltreatment experienced previous to living on the streets, influences prospects of SICY reintegration beyond institutional care facilities has also not been previously reported.

Crombach et al. (2014) found post-traumatic stress disorder mediated associations between previous traumatic experiences and class attendance at an institutional care facility in Burundi. Cultivating interests in activities and hobbies is recognized as a resilience-promoting practice, though it is unclear the extent to which this process may be mediated by the promotion of grit, self-esteem, self-efficacy, positive social identity, autonomy, self-regulation, or some other psychological trait (Gilligan, 1999; Howell, 2011). The degree to which SICY interests in activities on the street predict future reintegration prospects have not been reported within sub-Saharan Africa.

Finally, the KPJ/Flourishing Community model was developed to support families and communities from which SICY had left to live on streets. While the program shares features with other programs, we are unaware of any program integrating economic, social, health, and educational elements as does the KPJ program with the intention to support the reintegration of SICY (Goodman et al., 2020). Whether family participation in the KPJ, or similar, program is associated with reintegration prospects has not been reported within sub-Saharan Africa or other low- or middle-income contexts.

### 1.7. Study aim

This study aims to characterize post-interventional reintegration outcomes, and significant predictors, from multiple waves of SICY participants in the ARC program. Reintegration outcomes report structural location after exiting the ARC program. Assessed predictors reflect pre-street, street-, and post-street-life characteristics of the child, family, and community.

#### 1.7.1. Implied sub-aim

**FIGURE 4:**
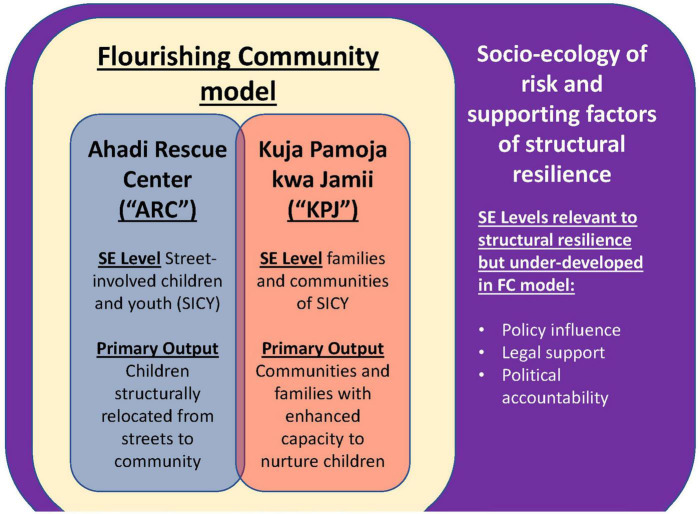
Socio-ecological levels of structural resilience risk and promoting factors for SICY within the Flourishing Community interventional model. SE level, socio-ecological level; FC, Flourishing Community model.

While the primary focus of this study is on immediate reintegration outcomes and significant predictors from the ARC program, we also evaluate whether participation in the KPJ program is associated with improved reintegration outcomes as intended. Figure 4 depicts theoretical relationships between two programs, an umbrella interventional model and the socio-ecological framework of structural resilience. An implied sub-aim of this study moves beyond immediate reintegration outcomes of SICY to assess validity of the hypothesized synergy between SICY-focused activities (the ARC program) and the community-based program to support families and communities of origin for SICY (the KPJ program). As depicted in Figure 4, these two programs, and the Flourishing Community model they inform, seek to improve structural resilience within a socio-ecological framework. This study is intended to lend support or nuance to this strategy.

## 2. Materials and methods

### 2.1. Study design

This is a retrospective cohort study, utilizing program data related to participants in the ARC program from multiple data sources. The study is set in Meru County, Kenya, using data from April 2016 through October 2022.

### 2.2. Data source

All data analyzed in this study were collected from program involvement with children and youth identified living on streets in three sub-counties of Meru County, Kenya between April 2016 and October 2022. Four forms were integrated into one dataset for present analyses – child intake, exit, follow-up and initial household interview. All available data from the program were digitized, and linked at the individual-level. The combined file was then deidentified to support statistical analysis.

Intake data were available for 253 instances. Of these, 25 were repeated from the same child who had relapsed to the streets following a previous intervention. The repeated observations were omitted from this study, utilizing just the first engagement and the child considered a “relapse case.” This left unique intake data from 227 children – 226 boys and 1 girl.

Of the 227 unique children, an initial household interview was conducted with families from 201 children. Sometimes it is not possible to identify a member of a child’s family because the child is a total orphan, or the parents have completely abandoned the child and moved without identifiable family.

Of the 227 unique children, 6 children lived at the ARC during the data digitalization and were omitted from the final analysis due to unknown outcome from the intervention.

Of the 221 unique children who had left the intervention, follow-up records were available for 127. Program staff report various characteristics influence the extent to which they are able to follow-up with previous program participants. Children who are in more secure environments are less likely to be followed up due to resource limitations, and children who live in more migratory environments are harder to be followed up due to falling out of contact.

#### 2.2.1. Intake form

Trained, paid social workers conduct routine walks through town streets in the catchment area, identifying children and youth who have moved to the streets and developing rapport. During recruitment periods, which occur when school is in session to identify young people who should be in school but are instead on local streets, social workers identify young people who report sleeping on local streets and express an interest in reintegrating with the broader community. During this period, SICY who report an interest in leaving street life are interviewed using a semi-structured questionnaire.

##### 2.2.1.1. Intake data overview

Street-involved children and youth provide information on their home village and family background, age, years on the street, motivation for migrating to the street, years in school completed, interest in activities (including sports, socializing, cooking/cleaning, and others), chores at home before moving to the streets, activities engaged in during life on the street, source of food, forms of abuse experienced on the street, general health, substances used on the street and desired outcome of engaging with the program. The date the child moved to the ARC, or was directly reintegrated, is included with the analysis. As each child is at the ARC under permission from a family member or member of the government, the intake form provides information about whether the child has identifiable family or not.

###### 2.2.1.1.1. Time of entrance to the program

Data reflect a series of cohorts through the ARC program, and the evolving, more personalized timing of coordinating activities around the need of each child rather than a standard preset duration of time (reported in section “Results”).

###### 2.2.1.1.2. Family background data

Street-involved children and youth provide information related to their family backgrounds, including whether their parents are living, deceased or unknown; who their primary guardian was at home; and how many living siblings they have.

#### 2.2.2. Initial household interview form

After securing temporary approval from the Child Protection Office to assume care for a child identified living on the street, social workers attempt to identify the family of the child who was brought into care at the Watoto Wa Ahadi Rescue Center – or directly reintegrated, if possible. When possible, family data are recorded and verified if previously provided by the child.

##### 2.2.2.1. Initial household interview data overview

Social workers engage with village chiefs and neighbors to identify families of origin reported by SICY. Whenever possible, social workers rely on the closest family available to provide information about the identified child. The data recorded by social workers include food and housing quantity and quality (good, fair, and poor), family challenge areas (including substance use, housing insecurity, relational stability, food or water security, and foster family), occupation and health of the parent, and whether the family owns land.

#### 2.2.3. Exit form

After working with the child and family to develop a reintegration plan, whenever possible, and staying at the ARC for long enough to meet remedial goals of reintegration, the child or youth is reintegrated with their home community or another option beyond the ARC (e.g., polytechnic school or financial support to start a business).

##### 2.2.3.1. Exit form data

Data regarding the child’s time at the ARC, including date and destination upon leaving the ARC are recorded on an exit form for each child. Services offered to the child, duration of time at the ARC, school participation, and whether the child’s family joined the KPJ program during his tenure at the ARC is recorded.

#### 2.2.4. Follow-up data

Upon reintegrating a child or youth with their destination post-ARC intervention, social workers rotate visitations with children and youth previously served by the organization and attempt to offer on-going counseling and referrals as necessary and possible to the children and their caregivers. This activity provides an opportunity to revise understandings of where the child is currently engaged – including if the child has returned to living on the streets. The date on the follow-up form provides an indication of how long the child persisted in the outcome documented on the exit form, though only cases where the child had relapsed were noted as different to the exit form data. Follow-up data were included to control for potential loss-to-follow-up confounding, and to inform follow-up-oriented resource utilization and strategy.

### 2.3. Analysis plan

This study is principally concerned with the placement of children after participating in the ARC intervention and predictors of the positive outcome of being reintegrated with a family and enrolled in school.

#### 2.3.1. Outcome variable

There were originally five potential outcomes of the program: (1) enrolled in school and living at home; (2) enrolled in school but not at home – e.g., boarding school; (3) enrolled in a polytechnical school to gain a skill or otherwise supported to start a business; (4) returned to the streets – either by running away from the program before finishing, or relapsing to the street after a reintegration attempt; and (5) still living at the ARC at the time of data digitization. To support multinomial logistic regression, these five outcomes were reduced to three – (1) with family and enrolled in school; (2) enrolled in boarding or polytechnical school, or supported to start a business, and (3) relapsed to the streets or fled the program prior to completion. Children who remained at the ARC at the time of data digitalization were excluded from analyses, as their outcome was not yet known.

#### 2.3.2. Predictor variables

The four data sources – intake form, exit form, follow-up form, and initial household interview – provided data that may be significantly associated with the defined outcome variable. These four sources provided information about different time points across the intervention’s relation to the child, and the child’s own history. These data were sorted into four different subsets for analysis according to theorized proximity to the outcome. Each subset was analyzed separately to identify variables that were significantly associated with the outcome before assessing retained variables in sequence from more distal to more proximal relation to the outcome. Statistically, it would not be possible to distinguish confounding, suppressing or mediating relations between variables, and there is insufficient theory and evidence to suggest probable pathways (MacKinnon et al., 2000).

##### 2.3.2.1. Four subsets of data

To support assessment of a large set of variables within an exploratory evaluation, we grouped data into groups based on timing and proximity of these variables to the outcome. The rationale for this approach was that more proximal variables may explain (mediate) associations between more distal variables and interventional outcome. Rather than ignore the potentially significantly associated distal variables, we included them in initial models before determining the final model.

###### 2.3.2.1.1. Home environment and background

Home environment and background data included: child’s current age; sub-location before migrating to the street; guardian status; child’s activities at home; years of school completed before migrating to the streets; child’s dislikes at home; health, and economic status of household; and whether any family member could provide consent for the child to spend time at the ARC center.

###### 2.3.2.1.2. Reported experience on the streets

Data reported by the child, and recorded on the intake form, relevant to the child’s time on the street were grouped and analyzed together, including: time on the streets, forms of abuse reported on the street, age when the child first migrated to the streets, interests and behaviors on the street, manner of securing food, sleep location, and substance use behaviors.

###### 2.3.2.1.3. Interventional characteristics – ARC

Characteristics of participating in the ARC program were assessed – including time spent in the program, date entering the program, child’s goals for life after the intervention, and duration of follow-up post-intervention. Duration of follow-up and date entering the program were sub-divided into five quantiles to facilitate interpretation.

###### 2.3.2.1.4. Interventional characteristic – KPJ

Kuja Pamoja kwa Jamii participation was included as a binary measure – the child has a family member enrolled in the KPJ program vs. the child does not/it is unknown.

#### 2.3.3. Modeling strategy

The outcome variable is described using proportion for each possible outcome – first with the five potential outcomes, and then as reduced to three outcomes. Multinomial logistic models were calculated to identify variables associated with moving back with a member of the family and attending school, compared to the other two options – enrolling in polytechnical school, boarding school, or starting a business; or returning to live on the streets during or after the ARC intervention.

Descriptive statistics are reported for all variables that were significantly associated with the multinomial outcome variable – for each subset of data, and for the final model. Variables within each subset of data were assessed simultaneously through multinomial logistic regression and were retained in the model for that specific data subset if they were significant at *p* < 0.20; the alpha threshold for this study was 0.05.

The final multinomial logistic regression model was created by including all variables significantly associated with the outcome for each of the four subsets of data and all retained variables that were significantly associated with either of the two outcome comparisons at *p* < 0.05.

#### 2.3.4. Ethical consideration

Initial data collection for the project was given ethical approval by the Children’s Office of Meru County, Kenya, with consent provided by proxy for care of each participating child until family contact could be established by the program. Each child provided assent to participate in the program, including data that is recorded at each phase of the project. Data were linked by program facilitators prior to being deidentified for used by researchers. The Institutional Review Board at the University of Texas Medical Branch provided ethical exemption for the analysis of secondary, deidentified program data.

#### 2.3.5. Data analysis software

All data were analyzed in STATA v.16.1 (StataCorp, 2019).

## 3. Results

### 3.1. Visualizing program exposures

**FIGURE 5:**
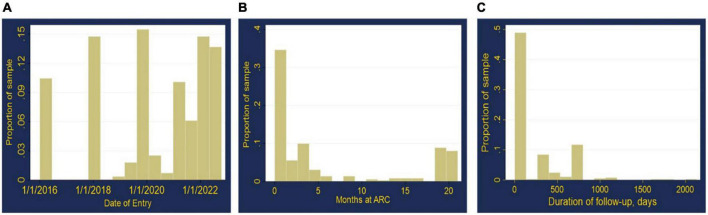
**(A–C)** Data of entry to ARC, months spent at ARC, and duration of follow-up among intervention subjects.

Figures 5A–C show (A) dates of enrollment, (B) months spent at ARC, and (C) duration of follow-up for all included children. Figure 5A demonstrates the increase in number of SICY supported through the program as the program transitioned from longer stay to shorter stay around January 2020. Figure 5B demonstrates the number of months children and youth have stayed at the ARC, with the largest number of SICY staying for less than 5 months. Figure 5C demonstrates the length of follow-up for each child.

### 3.2. Descriptive outcomes of sample

**FIGURE 6:**
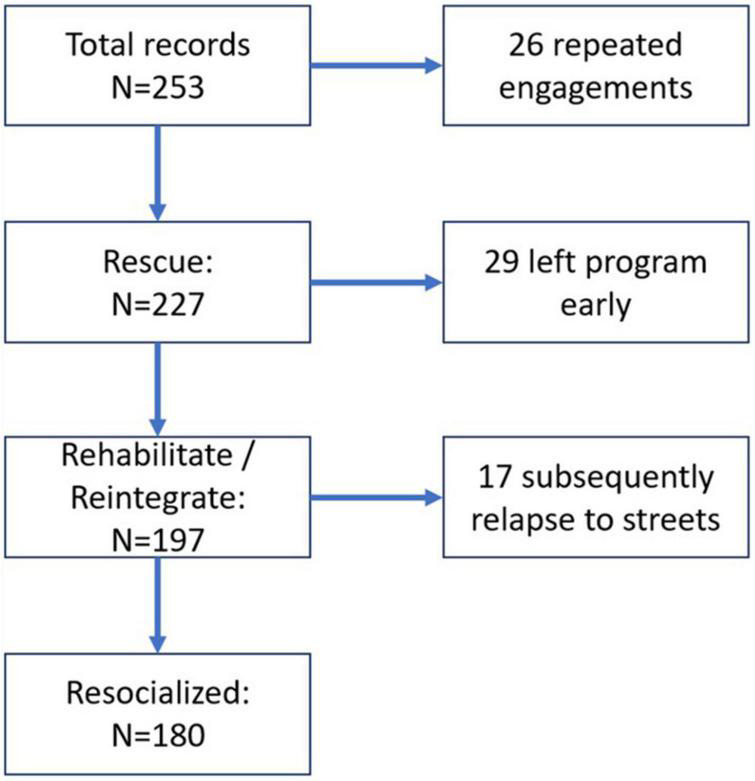
Structural interventional outcomes.

Table 3 displays variables included in the outcomes or associated with the outcomes in analysis of a limited or full regression models. Over 50% of children who previously lived on the streets were reintegrated with their families and returned to school, with an additional 8% placed in boarding schools, 14% placed in polytechnical schools, and under 3% starting a business. Nearly 13% of participants left the program early, and nearly 10% were known to have relapsed to the streets at least once after being reintegrated to the broader community. As shown in Figure 6, there were a total of 253 initial records, of which 25 were repeated engagements with the same children. Of the 227 SICY who were rescued from the streets at one point, 29 left the program early and returned to life on the streets. Of the 197 SICY who were rehabilitated and reintegrated to some other location, 17 subsequently relapsed to the streets, leaving 180 (of the original 227) who were reintegrated with follow-up.

**TABLE 3:** Univariate and bivariate description of model variables.

		Univariate			Outcome A		Outcome B		Outcome C		*p*-Value
		***N***	**Mean (%)**	**SD**	**Mean (%)**	**SD**	**Mean (%)**	**SD**	**Mean (%)**	**SD**	
Outcome A	Reintegrate with family and return to school	226	51.0%	0.4							
Outcome B: reintegrate someplace other than home	Boarding school	226	8.0%	0.3							
	Polytechnical school	226	14.1%	0.3							
	Start business	226	2.7%	0.2							
Outcome C: return to street	Leave program early	226	12.8%	0.3							
	Relapse	226	8.8%	0.3							
Censored from analysis	Still at ARC	226	3.0%	0.2							
Subset 1	Age	218	13	2.1	12.53	2.12	13.89	1.89	13.54	2.10	<0.001^Δ^
	Years of school	194	4.9	2.2	4.83	2.08	5	2.29	4.70	2.56	0.75^Δ^
	Family owns land	219	31%	0.46	35.04%	0.48	33.93%	0.48	17.39%	0.38	0.07^ε^
	No identifiable family at intake	219	21%	0.41	26.50%	0.44	7.14%	0.26	26.09%	0.44	0.006^ε^
Subset 2	Years on street	179	1.6	2	0.92	1.34	2.83	2.25	1.55	2.16	<0.001^Δ^
	Street abuse, emotional	219	37%	0.48	32.48%	0.47	51.79%	0.50	28.26%	0.46	0.02^Ω^
	Street abuse, economic	219	28%	0.45	23.08%	0.42	46.43%	0.50	17.39%	0.38	0.001^ε^
	Interest index (range: 0–3)	187	1.2	1.2	1.37	1.31	1.24	1.11	0.82	1.19	0.007^Δ^
Subset 3	Entry date to ARC (*q5*)	218	2.8	1.4	3.08	1.40	1.69	0.88	3.41	1.33	<0.001^Δ^
	Months spent at ARC	193	7.7	8.2	6.49	7.94	13.39	7.94	3.71	5.09	<0.001^Δ^
	Child desires to reintegrate with family	219	29%	0.45	37.61%	0.49	16.07%	0.37	21.74%	0.42	0.007^Ω^
	No follow-up	219	52%	0.5	44.8%	0.50	50.0%	0.50	67.4%	0.47	0.04^Ω^
	Follow-up duration^ψ^	107	530	358.4	509	282	652	489	395	318	0.23^Δ^
Subset 4	Family joined KPJ	219	12%	0.33	17.95%	0.39	8.93%	0.29	2.17%	0.15	0.01^ε^

Sample mean or percentage and standard deviation provided for model variables. Specific outcomes combined to create Outcomes A, B, and C presented. *p*-Value for different bivariate tests of independence provided.

^Δ^Kruskal–Wallis test.

^Ω^Chi-square.

^ε^Fisher’s exact.

^ψ^Follow-up duration includes only cases with any reported follow-up, and is reported in days between program exit and last reported follow-up contact.

#### 3.2.1. Comparison of variables across outcome strata

As shown in Table 3, the average child was 13 years old, and younger children were significantly more likely to be reintegrated with their families than another outcome. The average years of completed schooling was 4.9; age was not significantly different across outcome categories. Over 30% of children had families who owned land, which was 50% lower among children who relapsed or left the program early compared to children who were reintegrated to families of origin or another location. Over 1 in 5 children had family who could not be identified when they entered the program – arriving under government consent. Children who were reintegrated with a polytechnical or boarding school had the lowest percentage of children without identifiable family at intake. The mean years on the street (1.6 years; SD: 2) was significantly lower among children who were reintegrated with their families of origin than children who were in the other two outcome categories.

Over 35% of children reported being emotionally abused on the street, and over 25% of children reported being economically abused on the street. Reported experiences of emotional or economic abuse were significantly lower among children who left the program early or relapsed to the street. Among children who were reintegrated, experiences of abuse were lower among children who were reintegrated to their families of origin. The index of reported interests was significantly higher among children who reintegrated to their families of origin compared to children who relapsed or left the program early. Children who were reintegrated to families directly entered the program significantly later, on average, than children who were reintegrated to boarding or polytechnic schools or who began a business. Nearly 1 in 3 children reported at intake their goal was to reintegrate with their families, and this percentage was significantly higher among children who were reintegrated with their families or who returned to the streets. Over 50% of children were reported as without follow-up post-intervention, which was 23% higher among children who returned to the streets (67%) compared to children who were reintegrated with their families (45%). Among those who received reported follow-up visits by a social worker, the duration of follow-up was highest for children who were reintegrated to a boarding or polytechnical school. Only 12% of children had families who had joined the KPJ program, and this was highest among children who were reintegrated with their families (18%) and lowest among children who returned to the streets (2%).

### 3.3. Comparing outcomes across subsets of data

Table 4 shows the multinomial analyses of 4 data subsets.

**TABLE 4:** Multinomial logistic regression of program outcomes on four subsets of predictor variables.

	Subset 1						Subset 2						Subset 3						Subset 4					
**Outcome:**	**B**			**C**			**B**			**C**			**B**			**C**			**B**			**C**		
	**RR**	**95% CI**		**RR**	**95% CI**		**RR**	**95% CI**		**RR**	**95% CI**		**RR**	**95% CI**		**RR**	**95% CI**		**RR**	**95% CI**		**RR**	**95% CI**	
Child’s age (years)	1.47***	1.22	1.77	1.3**	1.09	1.55	1.40***	1.14	1.73	1.24*	1.04	1.47	1.59***	1.26	2	1.25*	1.05	1.49	1.41***	1.18	1.69	1.28**	1.07	1.53
Family owns land	0.63	0.3	1.31	0.33*	0.13	0.81																		
No identifiable family	0.16**	0.05	0.51	0.67	0.29	1.56																		
Years on the street							1.56***	1.26	1.95	1.13	0.88	1.47												
Abuse on streets, emotional							2.99*	1.26	7.11	0.56	0.24	1.29												
Abuse on streets, economic							2.62*	1.07	6.37	0.77	0.28	2.09												
Number of interests							1.12	0.79	1.6	0.63**	0.45	0.89												
Later entry date to ARC (q5)													0.27***	0.16	0.45	0.73	0.49	1.08						
Months spent at ARC													0.97	0.91	1.04	0.92*	0.84	0.98						
Child desires to reintegrate with family													0.89	0.32	2.43	0.4*	0.17	0.92						
No follow-up													4.15***	1.74	9.87	3.06*	1.23	7.62						
Family joined KPJ																			0.44	0.15	1.28	0.1*	0.01	0.77

Multinomial logistic regression comparing three outcome categories using two sets of comparisons: A vs. B, and A vs. C. (A) Reintegrated with family and enrolled in school; (B) placed in boarding school, polytechnic school, or supported start a business; (C) left program early, or subsequently relapsed. Four subsets reflect the four stages of (1) life before street, (2) life on street, (3) life in ARC program, and (4) program support received by family. *Indicates *p* < 0.05; **p < 0.01; ***p < 0.001.

#### 3.3.1. Families of origin and outcomes

The first subset shows older children were significantly more likely to be in Outcome B (reintegrate to boarding school, polytechnic school, or start a business) or Outcome C (return to streets) compared to Outcome A (reintegrate with family and return to school). Children without identifiable families at intake were more likely to be in Outcome B compared to reintegrated with their families of origin, before controlling for variables in other data subsets.

#### 3.3.2. Street experiences and outcomes

The second data subset assesses variables related to street experiences. Children who reported more years on the street had significantly higher rates of reintegrating someplace other than with their families of origin. Reporting emotional or economic abuse on the streets predicted significantly higher rates (2.99 and 2.62, respectively) of reintegrating some place other than families of origin. Children who reported more interests at intake were significantly less likely to return to the streets later.

#### 3.3.3. ARC program exposure and outcomes

The third data subset showed children who entered the program at a later date were more likely to reintegrate with their families of origin than children who reintegrated to a polytechnical school, boarding school, or start a business. Children who returned to the streets spent less time on average at the ARC and had less desire to reintegrate with their families of origin.

#### 3.3.4. KPJ program exposure and outcomes

The fourth analysis revealed that children who returned to the streets were significantly less like to have families who were in the KPJ program. Children who returned some place other than with their families of origin were also less likely to have families in the KPJ program, but this was not statistically significant.

### 3.4. Final model of outcomes

Table 5 shows the combined, final multinomial logistic model comparing Outcome A (reintegrating to families of origin and attending school) to Outcome B (reintegrating some place other than with families of origin) or Outcome C (returning to the streets).

**TABLE 5:** Multinomial regression of program outcomes on multi-level and multi-component elements.

	Dependent variable category					
	**Outcome B**			**Outcome C**		
**Description of category**	**Reintegrated some place other than family of origin**			**Returned to the streets**		
	**RR**	**95% CI**		**RR**	**95% CI**	
Intercept	0.1**	0	0.2	0.03*	0	0.5
Child’s age	1.53**	1.14	2.05	1.22*	1.03	1.45
Years on the street	1.3^t^	0.98	1.72	1.36^t^	0.94	1.96
Street abuse, economic and/or emotional	2.69**	1.24	5.82	0.81	0.38	1.74
Interests (sum)	1.96**	1.24	3.09	0.7^t^	0.46	1.05
Date of entry (q5)	0.28***	0.17	0.47	0.84	0.56	1.28
Child desires to reintegrate with family	0.3*	0.1	0.88	0.68	0.23	2
Months at ARC	0.96	0.9	1.03	0.91*	0.84	0.99
Family joined KPJ	0.79	0.14	4.4	0.12*	0.02	0.84
No follow-up	4.55**	1.76	11.75	2.73*	1.07	6.99

Multinomial regression with robust standard errors comparing (A) reintegration with family/attending school with (B) placed in boarding school, polytechnical school, or starting a business or (C) leaving program early/relapsing to streets. *p < 0.05; **p < 0.01; ***p < 0.001; ^t^*p* < 0.1.

Controlling for other factors, older children were significantly less likely to be reintegrated with their families of origin. Children who were on the street longer were less likely to reintegrate with their families of origin (*p* < 0.1). Children who reported experiences of emotional or economic abuse on the street were more likely to reintegrate someplace other than their families of origin. For each interest on the interest index reported by a child at intake, the rate of reintegrating to someplace other than families of origin were nearly doubled, and rates of returning to the streets reduced by 30% (*p* < 0.08). Children who entered the program at a later period were significantly more likely to be reintegrated with their families of origin than to another location. Children who expressed a desire to reintegrate with their families of origin at intake were significantly more likely to be reintegrated with their families of origin subsequently. Children whose families were in the KPJ program had significantly lower rates of returning to the streets, controlling for other factors. Children who were in the program longer had higher rates of reintegrating with their families of origin. Children who were reintegrated with their families had significantly higher rates of follow-up than children who were reintegrated some other place or returned to the streets.

## 4. Discussion

Through this analysis of program data, we intended to understand positive outcomes from a program intervention that embraces a socio-ecological perspective of children and youth living in street situations. Furthermore, we aimed to animate, inform and encourage application of structural resilience-oriented research and interventional work.

### 4.1. Program findings

As data showed, the program underwent an evolution in its practice around the time of COVID-19, and in part as a response to government shutdowns to control the pandemic. As such, the number of children who passed through the program increased substantially between January 2020 and the most recent entrant in October 2022 (Figure 5a, above). The original consideration for keeping children at the ARC program for 2 years was informed partially by considerations of how to identify families of origin, recruit them to the KPJ program, and establish solid relationships and preparations for reintegration of children. Despite this intention, the majority of children returned to families of origin and did so from later waves of recruited SICY. Given the intention, and likely necessity, to support families and communities of origin to provide better support to children returning home from street situations, identifying mechanisms to rapidly respond to the reintegration of SICY by developing social support systems for the children and their families is essential. As data show, participation in the KPJ program is significantly associated with children not returning to street situations. The KPJ program demonstrates rapid growth and acceptability, positioning the strategy to combine reintegration efforts with community transformation efforts as meriting further research and development. The fact that duration on the street predicts significantly lower rates of reintegrating with families of origin indicates the need for early intervention with children who newly arrive on the streets. We previously found duration of time on the street predicts substance use (Goodman et al., 2023), which is consistent with socialization in street culture that protects SICY by providing an alternative social habitus to the broader culture (Hills et al., 2016). Extended time on the street may reinforce participation and identification with a sub-culture in opposition to the broader culture and may challenge any existing bonds of affection between SICY and their families. Rapid intervention appears necessary to promote family-based reintegration, requiring further shifts in community-based programming.

#### 4.1.1. Children’s self-determination and mental health

Children’s interests/desires at time of intake predicted subsequent outcomes. Children who expressed a desire to reintegrate with their families were more likely to do so. Children who expressed fewer interests in any activity (e.g., sports or socializing) prior to being admitted to the ARC were more likely to return to the streets subsequently. Lack of interest in activities is characteristic of depressive symptoms, but could be due to other socio-ecological or psychological factors. Mental health states conducive to successful reintegration should inform future research efforts, including depression, hope, and psychological resilience (e.g., Watson et al., 2020; Lenz, 2021).

The ARC program began collecting psychometric data on children entering the program in April 2016, but program leadership abandoned this approach until greater clarity could be gleaned to inform what psychometric properties were likely to be important. From these observations, we find a few different measures that may be important to promoting structural resilience.

#### 4.1.2. Potential post-traumatic stress, blame attribution, and depression

Children who reported more abuse on the streets were less likely to reintegrate with their families of origin, controlling for an expressed desire to do so. This may be related to persistent PTSD, lack of sense of felt safety, or other psychosocial factors (Morton et al., 2022; Neuner, 2022; Wesarg et al., 2022).

Lingering traumatic experiences may complicate integration of children with families and communities of origin and may drive children to attribute their street-based hardships to their families of origin (Shaver, 2012). The extent to which these dynamics undermine family-based reintegration of SICY is unexplored to our knowledge. Depression, marked by a lack of interest, may also prevent children from wanting to envision alternatives to their street situations. Measurements of PTSD, attribution of guilt, felt safety, and depression may inform structural resilience initiatives with SICY (Derivois et al., 2017). Further, understanding how SICY form and express values, goals, and interests, and how to promote prosocial values and goals, may be a generative direction for structural resilience interventions.

#### 4.1.3. Secondary findings

While excluded from primary analyses, 22 observations from the original total 253 engagements were from the second encounter with children and three observations were from the third engagement with children. Of the 22 second-time encounters, 8 (36%) returned home and to school, 3 (14%) returned to a polytechnical or boarding school, 9 (41%) relapsed to the streets or left the program early, and 2 (9%) remained at the ARC at the time of data entry. Of the 3 third-time encounters, 2 returned to the street and 1 remained at the ARC at the time of data entry.

#### 4.1.4. Protection on the streets

In addition to addressing the mental and behavioral health of SICY to support family-based reintegration, interventions properly informed by a socioecological perspective will seek to promote safety of children living on the streets. This must not be a final strategy, but rather a harm-reduction approach to support reintegration of children whenever possible. In presenting these findings to local stakeholders in Meru County, leaders of the local police force brought up that the forms of abuse reported by SICY in the intake form were criminal. The ARC program strengthened its relationship with the police leadership with a commitment to report these cases and work with the legal system whenever possible. However, the ARC leadership also noted that children report being mistreated by police on the streets too, undermining their confidence in the protection the police might provide. Finding workable solutions to this tension between trust and mistrust of police and other adults, while ensuring the rights of children living on the street, requires locally contextualized approaches. Reducing harm toward SICY will require engaging with community norms and attitudes toward people living on the street – regardless of age. Normative engagement and stigma reduction is supported by the Flourishing Community model (Goodman et al., unpublished) but requires further development to protect SICY.

#### 4.1.5. Suggested directions

##### 4.1.5.1. Data standardization

In the context of data collection for reintegrating SICY, data gaps were treated as “0s” due to limitations in the original paper forms. These forms did not provide a way to differentiate between negative responses and missing data (e.g., unanswered questions). Standardizing intake, exit, follow-up and household interview questionnaires is a requirement for successful implementation of Kenyan and international policy shifts toward reintegrating SICY. Treating missing variables as all null was the most conservative approach, decreasing likelihood of rejecting null hypotheses.

##### 4.1.5.2. Family follow-up

The 4R + P strategy, which combines street-based outreach with family and community-level transformation, is shown to be effective. However, further research is needed to understand the characteristics of responsive, adaptive, and welcoming families and communities for reintegrating SICY. While family participation in the program is associated with lower rates of returning to the streets, other factors need to be considered. It is important to determine the factors influencing participation in the program and the long-term outcomes of reintegrated children. Additionally, research is needed to assess the improvement in developmental, psychosocial, and other domains among children whose families participate in the program. Anecdotal evidence suggests that communities provide economic, social, educational, and food support for KPJ families and welcome returning children back into the community. However, further research is required to understand the adaptation of these children and the factors that support successful reintegration in educational, psychosocial, physical, and future economic domains.

##### 4.1.5.3. Active monitoring and follow-up

Policy shifts regarding the work with SICY, both in Kenya and globally, have been driven by a recognition of the exploitative depictions of institutionalized children for fundraising purposes, which often result in limited benefits for the children themselves. To prevent a repetition of such ineffective practices, active monitoring is crucial. We included post-intervention monitoring as a variable to address potential censoring and to guide the strategic use of follow-up measures. However, we found significant variations in the duration of follow-up across different outcomes. The original aim of the KPJ program was to support observations and follow-up of reintegrated children, but only 12% of families joined the program, hindering this effort. To ensure that children truly benefit and to prevent interventions from becoming mere revolving doors, wasting donor funding and community goodwill, it is imperative to make future investments in post-intervention follow-up. This will help to avoid children returning to the street situations from which they were supposedly “rescued.”

### 4.2. Structural resilience-related discussion

We advocate for the adoption of structural resilience as a determinant of other forms of resilience for SICY. Exclusively individualistic notions of resilience are inadequate and potentially harmful. “What makes it possible for this child to endure separation from adults, and constant exposure to emotional, physical, sexual, and economic exploitation and deprivation better than other children?” is akin to asking what enables Black Americans in Tuskegee, Alabama to better endure untreated syphilis when a treatment is available (Freimuth et al., 2001).

Supporting structural re-location, such as living with nurturing families, is an essential part of caring for children living on the streets. Program data show that it is possible to reintegrate SICY with their families of origin – here more than 50% of the time. While this study focused on structural outcomes, there is a need to assess other measures of well-being, including academic, mental, and social aspects. Global consensus and empirically supported best practices are urgently needed to enhance the resilience of SICY. Structural resilience provides an over-arching framework to consider other constructs of resilience that may benefit SICY (Southwick et al., 2014). There is urgent need for global consensus on measures and empirically supported best-practices to increase structural, educational, social, mental, behavioral, and physical resilience of SICY.

In the future, we will assess the predictive validity of transdiagnostic, integrative measures that synthesize multiple socio-ecological levels, sectors and processes – such as the Child and Youth Resilience Measure, and the Process-Based Assessment Tool (CYRM-28; Van Rensburg et al., 2019; PBAT; Sanford et al., 2022). The urgency of the need for such consensus within the complexity of the situation animates our preference for transdiagnostic, multilevel, and process-based measurements.

Working toward structural resilience requires robust theory- and evidence-informed socio-ecological frameworks to inform multi-level (individual, interpersonal, communal, institutional, and policy) actions to address the complex phenomenon of SICY. Multidisciplinary inputs and multisectoral cooperation are imperative to meet this opportunity to build a better world with these children and youth.

### 4.3. Limitations

The data analyzed in this study come from an active program working to reintegrate SICY and promote structural resilience. Data protection measures were limited, and findings should be assessed with caution. This study provides a glimpse of what is possible in intervention and policy-work with SICY and offers an orientation for future research. It is the first study assessing reintegration outcomes of an intervention with SICY in low- or middle-income countries. More studies are needed. The data analyzed in this study emerge from an active program working to reintegrate SICY and promote structural resilience. Data were not collected with the intention to support this study, and data protection measures were limited to self-imposed practices of a novel and evolving non-governmental organization. This study provides a glimpse of what is possible in intervention and policy-work with SICY and offers an orientation for future research. We believe this is the first study to quantitatively assess reintegration outcomes of an intervention with SICY in low- or middle-income countries. More studies are needed. The limitations in data collection and verification processes may have minimal overall impact if this study provides a blueprint for designing socio-ecological models and structural resilience with SICY.

## 5. Conclusion

This groundbreaking study provides the first evidence from sub-Saharan Africa, as well as any low- and middle-income country, that family-based reintegration of SICY is indeed possible. With over 50% of program participants (*n* = 227) successfully reintegrating with their families of origin, it demonstrates the potential for positive outcomes. However, it is important to note that over 20% of participants did return to live on the streets.

Several factors were identified as predictors of family-based reintegration, including younger age, fewer years spent on the streets, fewer experiences of abuse while living on the streets, the child’s desire to reintegrate with their family, and the involvement of a family member in a novel community-transformation intervention.

The implementation of new national and international policies regarding the reintegration of SICY requires significant shifts in programmatic design and intervention support. It is crucial that future research is guided by psychosocial, community-process, and intra-individual perspectives on resilience. However, this should be done within the framework of a larger socio-ecological view of resilience, referred to as “structural resilience” in this study.

In conclusion, this study provides valuable insights into the potential for family-based reintegration and calls for a comprehensive approach to addressing the needs of SICY in low- and middle-income countries.

## Data availability statement

The raw data supporting the conclusions of this article will be made available by the authors, without undue reservation.

## Ethics statement

The studies involving humans were approved by the University of Texas Medical Branch, IRB. The studies were conducted in accordance with the local legislation and institutional requirements. Written informed consent for participation was not required from the participants or the participants’ legal guardians/next of kin in accordance with the national legislation and institutional requirements.

## Author contributions

MG led the conceptualization of the study, statistical analyses, and wrote the original draft. SS provided additional statistical support, revision of subsequent drafts. AS provided the revision and editorial comments on each draft. AE provided the data curation and supported the field investigation. CM provided the final review edits. HS supported the data interpretation and policy analysis. PK and BR supported the funding acquisition and project conceptualization. LR-G contributed to project supervision, draft reviews, and interpretative analysis. CG contributed to interventional design and data curation. KM led data curation and draft review. SG contributed supervision and project conceptualization. All authors provided support for the conceptual development of the manuscript and final approval.
